# MICAL2 Promotes Proliferation and Migration of Glioblastoma Cells Through TGF-β/p-Smad2/EMT-Like Signaling Pathway

**DOI:** 10.3389/fonc.2021.735180

**Published:** 2021-11-12

**Authors:** Bei Pu, Xu Zhang, Tengfeng Yan, Yuntao Li, Baohui Liu, Zhihong Jian, Omer Kamal Mahgoub, Lijuan Gu, Xiaoxing Xiong, Ning Zou

**Affiliations:** ^1^ Department of Neurosurgery, Renmin Hospital of Wuhan University, Wuhan, China; ^2^ Department of Neurosurgery, The Affiliated Huzhou Hospital, Zhejiang University School of Medicine (Huzhou Central Hospital), Huzhou, China; ^3^ Central Laboratory, Renmin Hospital of Wuhan University, Wuhan, China; ^4^ Department of Radiation Oncology, Hubei Cancer Hospital, Tongji Medical College, Huazhong University of Science and Technology, Wuhan, China

**Keywords:** MICAL2, TGF-β, EMT, proliferation, migration

## Abstract

Recent studies showed that molecule interacting with CasL2 (MICAL2) could be a novel tumor growth factor, and it is closely associated with tumor growth and invasion. However, the role it plays in glioblastoma (GBM) and its potential mechanisms are currently unknown. Our study is designed to identify the effect of MICAL2 on GBM cells and the potential mechanisms behind it. Here, we found that MICAL2 interacts with TGF receptor-type I (TGFRI) and promotes the proliferation and migration of glioblastoma through the TGF-β/p-Smad2/EMT-like signaling pathway. MICAL2-knockdown inhibited the proliferation of glioblastoma cells, which was related to cell cycle arrest and downregulation of DNA replication. The invasion abilities of U87 and U251 cells were reduced after the knockdown of MICAL2. MICAL2 promoted the growth of GBM in nude mice. High MICAL2 predicts poor outcome of GBM patients. MICAL2 could be identified as a novel promising therapeutic target for human GBM.

## Background

Gliomas are the most common tumors of the central nervous system. Glioblastoma (GBM) is one of the most lethal cancers for which there is currently no ideal treatment. It accounts for 70%–75% of all diffuse gliomas, and the median survival of patients is only 14–17 months (1). Patients with primary gliomas do not have a documented history of lesser malignancy, and most patients with primary GBMs are the elderly. In contrast, GBMs that develop from low-grade gliomas (WHO grades I and II) are called secondary glioblastomas and are most commonly seen in younger patients under 45 years of age. These two have different molecular pathways of progression, with a critical determinant being mutations in isocitrate dehydrogenase 1 (IDH1). Its ability to rapidly proliferate and invade surrounding tissues is a significant cause of death in glioma patients. The traditional treatment of GBM is to remove the neoplasm by surgery, followed by chemotherapy and radiotherapy. Unfortunately, a complete resection of the tumor is not achievable due to the high invasive ability of GBM. Targeted therapy for glioblastoma also leaves an unsatisfactory treatment effect due to redundant compensatory mechanisms, the inability to cross the blood-brain barrier, or poor tolerability and safety (2, 3). Studies revealed that GBM cells could secrete multiple invasion-related proteases and migrate along meninges, the basement membrane of blood vessels, and white matter tracts (4, 5). However, the underlying mechanism of the migration and invasion of GBM still warrants further investigation.

Epithelial-to-mesenchymal transition (EMT) is a mechanism associated with epithelial tumor progression, invasion, and metastasis, in which polar epithelial cells eventually transfer to phenotypic mesenchymal cells. After undergoing morphological and biochemical changes, epithelial cells could acquire a fibroblast-like morphology to migrate from the epithelial layer (6). Meanwhile, epithelial cell-cell interaction and cell-basement membrane adhesion are reduced to alter the polarity of a cell and boost cell migration, and it is a crucial step in tumor invasion and metastasis (7). Moreover, downregulation of E-cadherin expression is the most significant marker alteration of EMT in an epithelial tumor. Different from typical epithelial cells, neural cells present a mesenchymal phenotype. While there is a transformation process similar to EMT during the progress of GBM, which is called EMT-like process (8). The EMT-like process mainly represents the decrease of epithelial markers such as E-cadherin and the increase of interstitial markers such as N-cadherin and vimentin (9). A study portrayed that the overexpression of a “mesenchymal” gene expression signature is linked to a poor prognosis of glioma patients (10), indicating that the EMT-like process is closely associated with the invasion of GBM. Some regard the EMT, cancer stem cells (CSCs), and drug resistance as the lethal “three combinations” and have become the main barriers for glioma to be cured (11). The inhibition of EMT may prevent invasion and metastasis of tumor cells, reduce CSCs, and overcome drug resistance.

One primary signaling pathway to stimulate the EMT is the transforming growth factor-beta (TGF-β) signaling pathway (12). TGF-β is a multifunctional cytokine that acts as a bidirectional regulator in the progression of the tumor. In the early stage of tumorigenesis, TGF-β serves as a potential tumor suppressor. As the tumor progresses, TGF-β is converted to a tumor-promoting factor, promoting EMT and tumor metastasis (13). TGF-β can mediate both the classical Smad signaling pathway and the nonclassical Smad-independent signaling pathway. TGF-β elicits its biological activity through dimerization by hydrophobic interactions, and it binds to paired TGF receptor-type I (TGFRI) and receptor-type II to form heterotetrameric receptor complexes (14). The classical Smad signaling pathway activation occurs when TGF-β first binds to the extracellular segment of TGF receptor-type II. Its intracellular segment Ser/Thr kinase is activated, which in turn leads to phosphorylation of TGF receptor-type I and subsequently activates the downstream Smad2/3 protein. The phosphorylated Smad2/3 recruits Smad4 to deliver the signal intracellularly.

In addition, TGF-β-activated non-Smad signaling pathways also play an essential role in tumorigenesis development (15–17). The current study displays that TGFRI/SMAD and its downstream signaling pathways perform a vital role in EMT. Smad2, Smad3, and Smad4 can form a complex that directly binds to the region of the Snail1 promoter and consequently promotes the expression of Snail1 (18). The Smad complex not only induces the expression of Snail1 protein but also binds to Snail1 protein and represses the transcription of various genes such as E-cadherin (19). Several studies have shown that the TGF-β signaling pathway participates in tumorigenesis and metastasis in advanced glioma, indicating that the TGF-β signaling pathway may be a promising target for glioma therapy (20–22). However, the potential molecular mechanism of the TGF-β signaling pathway promoting the invasive and metastatic ability of glioma cells still calls for further investigation.

The molecule interacting with CasL 2 (MICAL2) belongs to the MICAL family of proteins. It was first discovered by Suzuki et al. in 2002 (23). The MICAL family is a collective of actin-regulated redox enzymes often involved in the binding and disassembling of F-actin. It can be found in tissues rich in actin, such as the nervous system, skeletal muscles, and cardiac muscles (24). MICAL2 is widely expressed in various cancers, including glioblastoma (25), prostate cancer (26), bladder cancer (25), etc. MICAL2 participates in cell growth, cell apoptosis, axon guidance, and the transportation of vesicles by controlling the assembly and disassembly of actin (26, 27). Moreover, the MICAL2 family participates in metastasis and cancer cell invasion *via* the Sema pathway and increases the generation of reactive oxygen species (28). Besides, MICAL2 can also regulate cellular EMT through the SRF/MRTF-A signaling pathway, ultimately contributing to the accumulation of MRTF-A in the nucleus (29). The accumulation of MRTF promotes the ability of lung colonization and migration as well as inhibiting the migration of poorly metastatic tumor cells (30). However, the role of MICAL2 on glioblastoma progression and the mechanisms behind it are still unknown.

In our study, MICAL2 was widely expressed and coexpressed with TGF receptor-type I (TGFRI) in glioblastoma and was positively correlated with the expression of p-Smad2, which lies downstream of TGF-β (31). Furthermore, a negative correlation was discovered between MICAL2 expression level and the prognosis of patients with GBM. Further experiments revealed that MICAL2 could promote the growth of glioblastoma cells both *in vitro* and *in vivo*. Moreover, MICAL2 interacts with TGFRI and promotes the proliferation of glioblastoma cells through the EMT pathway. Mechanistically, MICAL2 promotes the proliferation and migration of glioma cells through the TGFRI/EMT signaling pathway. Understanding the molecular mechanism of MICAL2 participating in the invasion and metastasis of glioma cells can help us elucidate the molecular basis of the disease. Most importantly, MICAL2 could be identified as a novel promising therapeutic target for human GBM.

## Materials and Methods

### Immunohistochemical Staining

Immunohistochemical staining was performed according to the instructions of the manufacturer (immunohistochemical kit, MXB Biotechnologies, Fuzhou, China). Briefly, pathological sections were deparaffinized in a 65°C incubator, rehydrated by ethanol of different concentrations, and then antigen-retrieved with citrate solution. Next, endogenous peroxidase was inhibited by incubation with 3% hydrogen peroxide, closed with normal goat serum, and incubated overnight with the following primary antibodies: anti-MICAL2, anti-p-Smad2, and anti-TGF-β. After PBS rinsing, the sections were incubated for 20 min at 37°C with secondary antibodies and horseradish labeling of streptavidin. The slices were then rinsed and stained with DAB solution, and the nuclei were stained with hematoxylin. Finally, the slices were dehydrated and sealed with transparent glue and cover glasses. All samples were photographed with an inverted microscope (Olympus Corporation, Shinjuku, Japan). The relative expression levels of MICAL2 and p-amad2 were quantified using ImageJ.

### Assessment of Immunohistochemistry Results

Regarding the diffusivity of staining, the stained areas of the sections were graded as follows: 0, no staining; 1, <25% of the stained area; 2, 25% to 50% of the stained area; 3, 50% to 75% of the stained area; and 4, >75% of the stained area. As for the staining intensity, the sections were graded as follows: 0, no staining; 1, weak but detectable staining above control level; 2, significant staining; and 3, intense staining. The total IHC score was obtained by adding the diffusivity and staining intensity scores. A score <1.5 was classified as negative staining in cancerous tissue in patient samples, while a score >1.5 was classified as positive staining.

### Cell Lines and Cell Culture

The U87 and U251 cell lines were obtained from Shanghai Genochem (Shanghai, China). Cells were cultured in Dulbecco’s modified Eagle’s medium with a mixture of 10% fetal bovine serum and 100 U/ml penicillin-streptomycin. Cells were incubated in an incubator at 37°C and 5% CO_2_. U87 and U251 cells were treated with TGF‐β (5 ng/ml) for 48 h. Mycoplasma contamination was routinely detected. DMEM was purchased from Life Technologies (Carlsbad, CA, USA) and Gibco (Waltham, MA, USA); 0.25% trypsin was purchased from Life Technologies.

### Plasmid Construction

According to the coding sequence region of the *MICAL2* gene and previous studies (32), we designed and synthesized a pair of short hairpin RNA (shRNA) sequences. Plasmid (GV493-sh) expressing shMICAL2 was synthesized by Shanghai Genechem (Shanghai, China). The GV493-shMICAL2 sequence is shown in **Supplementary Table S1**.

### Cell Transfection

When the cell density reached 70%, cell transfection experiments were completed using Lipofectamine 2000 and oligonucleotides (40 μmol) according to the protocol; 5 × 10^4^ cells of U87 and U251 cell lines were transfected with 2 μg DNA of GV493-shMICAL2 or GV493-shscramble. The GV493-shscramble group served as a control. MICAL2 combination formula and scrambling control were obtained by puromycin selection. Transfected cells were collected 48 h after the transfection process, and then the transfection efficiency was verified by Western blot.

### Western Blotting and Immunoprecipitation

Total protein was extracted using a cocktail of RIPA lysis buffer, protease, and phosphatase inhibitor. We centrifuged the obtained cell lysates, and the concentration of protein in the supernatant was measured using spectrophotometry. Forty micrograms of protein was extracted and mixed with the loading buffer, and Western blot was performed. Proteins were separated by SDS-PAGE and then electrophoretically transferred to a PVDF membrane. After being blocked with 5% BSA, the membrane was incubated with a dilution of the primary antibody in a 4°C shaker overnight, then washed and incubated with the secondary antibody at 37°C for 1 h. We used the Odyssey Western blot analysis system to capture the image. The relative band intensity was normalized by the GAPDH control. We further used anti-MICAL2-agarose or anti-TGFRI-agarose antibodies to immunoprecipitate the clarified supernatant. MICAL2-immunoprecipitated complexes were identified using an anti-MICAL2 antibody.

### Tumor Growth Assays *In Vivo*

The tumor model was produced as previously described (33). BABL/c nude mice were obtained from the animal center of Shanghai Slac (Shanghai, China). MICAL2 knockdown cell line cells and the scrambled control were stereotactic injected into the left striatum, coordinates as described previously (33). The mice were divided into the MICAL2 knockdown and scrambled control groups according to the injected cells. Each group consisted of 15 mice. The tumors were monitored by luciferase visualization on the 7th and 21st days after implantation. In the second and fourth weeks, we used an MRI test to measure tumor size. At the first and third weeks, tumor tissues from nude mice were removed for HE staining and observed by microscopy.

### EdU Incorporation Assay

We used the EdU assay (KeyGEN BioTECH, China) kit to measure cell proliferation. After adding EdU working solution to the medium, we added EdU fluorescent molecules from the DNA synthesis process to make the proliferating cells fluorescent. After 48 h of transfection, we incubated the cells for another 24 h. Then, we treated the cells with EdU. Next, we fixed the cells in 4% formaldehyde and incubated them with 0.5% Triton X-100 at room temperature. After washing with PBS, the cells were reacted with a 1× Click-iT reaction mixture. Subsequently, the cells were stained for 15–30 min using Hoechst 33342. Finally, the nuclei were observed under a Nikon microscope after PBS washing (Nikon, Minato, Japan).

### Flow Cytometry Analysis

A flow cytometer was used to examine the cell cycle distribution. After 1 min of trypsin digestion, a single-cell suspension was obtained. The cell suspension was centrifuged at 1,000 rpm for 5 min, and the supernatant was discarded. Next, 500 µl of PBS solution containing RNase A (20 µg/ml) was added to the suspension and incubated at 37°C for 30 min, and the resulting supernatant was discarded by centrifugation. Next, wash them three times with 1 ml PBS. After washing with PBS and centrifuging at 1,500 rpm for 5 min, 500 µl of PBS solution containing PI (BD Bioscience, Franklin Lakes, NJ, USA) was added, and the resulting solution was incubated for 30 min at room temperature protected from light. Finally, the cells were analyzed using the EPICS ALTRA Flow Cytometer (Beckman Coulter, Brea, CA, USA).

### Immunofluorescence Analysis

Cells were incubated in 100% methanol (frozen at −20°C) for 5 min at room temperature, washed three times with PBS, and then the samples were incubated in PBS (with 0.1%–0.25% Triton X-100) and with 5% bovine serum albumin for 1 h. After washing three times with PBS, they were incubated at 4°C overnight with anti-MICAL2 and anti-TGFRI. Finally, the cells were incubated with a sufficient 300 nM DAPI staining solution for 1–5 min. They were observed under an automatic fluorescence microscope.

### Wound-Healing Assay

Cell migration experiments of glioma cells involve the re-colonization ability of MICAL2 knockout cells compared with control cells. Cell lines were cultured in six-well cell culture clusters and allowed to fuse to 80%. Then, we created a regular cross-wound with a pipette tip (1,000 µl). The surface was washed with PBS three times. The plates were incubated at 37°C and 5% CO_2_. After 24 h, the width of the scratches was observed and measured under the microscope, and a picture of the wound was taken.

### Transwell Migration Assay

When the cell density was greater than 80%, we used an 8.0-μm pore-size transwell analyzer (Corning, Corning, NY, USA) for transwell migration analysis. The transwells were constructed in a 24-well plate (Corning, Corning, NY, USA). First, 1 × 10^5^ tumor cells (U87 and U251) were added to the transwell in contact with the medium at the bottom of the well. Next, 300 µl of the resuspension was taken into the upper chamber for inoculation; 500 µl of the medium was then added into the lower chamber and incubated in a 24-well plate at a constant temperature for 48 h. Glioma cells that migrated downward were stained with crystal violet for 3 min. The air-dried chamber was then replaced with a washed 24-well plate, and the number of cells crossing the microporous membrane in five fields was randomly counted under a ×100 microscope.

### GEPIA Dataset

GEPIA (http://gepia.cancer-pku.cn/) is a web-based tool that provides services based on RNA sequencing data from The Cancer Genome Atlas and the Genome Tissue Expression databases. GEPIA provides interactive and customizable functions, including differential expression analysis, mapping, correlation analysis, patient survival analysis, and dimensionality reduction analysis (34).

### Statistical Analysis

For all statistical analyses, we utilized the GraphPad Prism 8.1 software (San Diego, CA, USA). Each experiment used an unpaired *t*-test or one-way analysis of variance, and a two-tailed Student’s *t*-test was used to compare individual groups. Experimental results were expressed as mean ± SEM. The statistical significance was set to *p* < 0.05. *p* < 0.01 and *p* < 0.001 represented a significant difference. Statistical analysis was performed on each data.

## Results

### MICAL2, TGF-β, and p-Smad2 Were Widely Expressed in High-Grade Glioma Cells at Higher Levels, and There Was a Positive Correlation Between MICAL2 and p-Smad2

**Figure 1:**
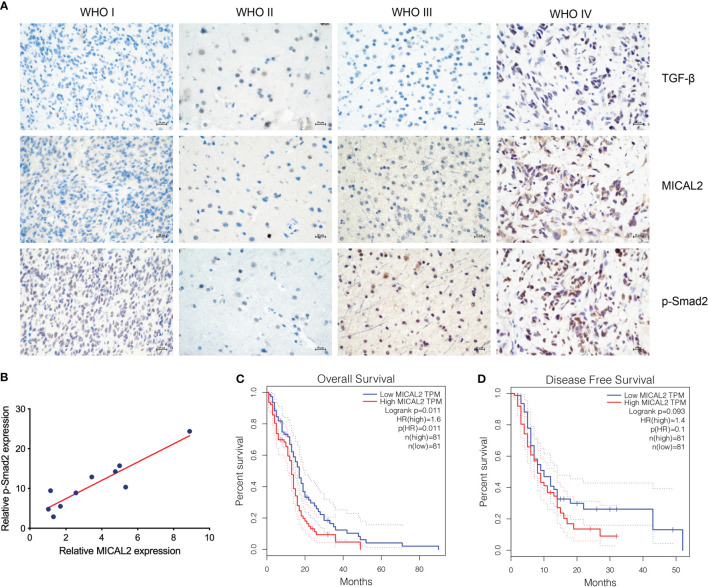
MICAL2, TGF-β, and p-Smad2 expresssion levels are elevated in GBM cells. **(A)** Immunohistochemical results showed that the expression level of MICAL2, TGF-β, and p-Smad2 are elevated in high-grade gliomas. **(B)** Regression linear equations fitted to the relative expression levels of MICAL2 and p-Smad2 in GBM tissues. **(C, D)** Kaplan-Meier survival analysis in 81 primary GBM cases stratified by MICAL2 expression level indicates that high level of MICAL2 expression was associated with lower OS (*p* = 0.011 < 0.05), but there was no significant difference in DFS between low MICAL2 and high MICAL2 expression levels (*p* = 0.093 > 0.05).

The expression of TGF-β, MICAL2, and p-Smad2 in different-grade gliomas was analyzed using immunohistochemistry. The immunohistochemical result showed that the expression of TGF-β, MICAL2, and p-Smad2 was increased with the grade of glioma (**Figure 1A**). The relation between MICAL2 and p-Smad2, which was a product of TGF-β downstream (31) was analyzed by multiple linear regression analysis. The results showed that there was a positive correlation between MICAL2 and p-Smad2 (**Figure 1B**). We then evaluated the impact of MICAL2 expression on the prognosis of patients with primary glioblastoma using data obtained from the GEPIA. High MICAL2 expression was recognized as a risk factor for overall survival (*p* = 0.011 < 0.05) in patients with primary glioblastoma by log-rank test (**Figure 1C**). However, there was no significant difference in disease-free survival between patients with high MICAL2 and low MICAL2 expression levels (**Figure 1D**).

### Downregulation of MICAL2 Inhibits Proliferation of GBM Cells *In Vitro*

**Figure 2:**
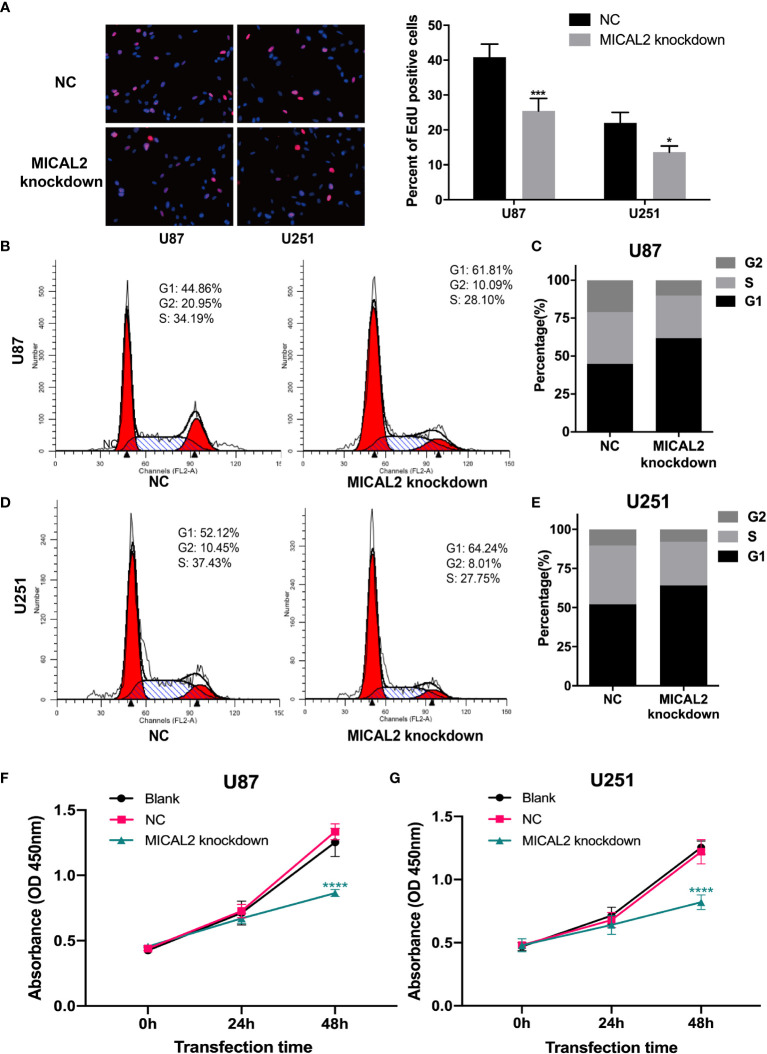
MICAL2 promotes proliferation of glioma cells *in vitro*. **(A)** EdU incorporation assay was used to detect U87 and U251 cell proliferation. There was a significant decrease of EdU-positive cells in U87 (*p* < 0.001) and U251 (*p* < 0.05) MICAL2 knockdown cells. **(B–E)** Cell cycle of U87 and U251 cells were analyzed *via* flow cytometry. Analysis of the cell cycle of U87 and U251 MICAL2 knockdown cells revealed a decrease in the proportion of cells in G2 and S phases. **(F, G)** CCK-8 assay demonstrated that inhibition of MICAL2 gene expression suppression had a significant inhibitory effect on glioma growth. Data are shown as the means ± SEM of three experiments. ^*^p < 0.05, ^***^p < 0.001, ****p < 0.0001.

To examine the biological significance of MICAL2 in GBM cells, we knocked down MICAL2 in both U87 and U251 cell lines using RNAi. We used the EdU assay to assess the impact of MICAL2 on cell proliferation. EdU can be incorporated into cellular DNA, replacing thymidine (T) during DNA replication (35). The number of U87 cells incorporating EdU in the MICAL2-knockdown group has decreased substantially (*p* < 0.001), when compared with the control group. Similar results also observed in U251 cells (*p* < 0.05) (**Figure 2A**). We then investigated the cell cycle distribution of GBM cells to further explore the effects of MICAL2 on the growth of GBM cells. Compared with the NC group, analysis of the flow cytometry showed that the MICAL2-knockdown U87 (**Figures 2B, C**) and U251 (**Figures 2D, E**) cells led to an increase in the number of cells during the G1 phase and a decrease in cell populations during S phase and G2 phase. Flow cytometry results indicated that the G1/S transition was hindered and the proliferation of GBM was inhibited by MICAL2-knockdown. Besides, CCK-8 assay demonstrated that inhibition of *MICAL2* gene expression suppression had a significant inhibitory effect on cell growth (**Figures 2F, G**). The expression of MICAL2 at each time point for both cell models was also detected using Western blot assay (**Supplementary Figure S1**).

### MICAL2 and TGFRI Affect the Mesenchymal Transition of Glioblastoma Cell and Promotes Migration and Invasion of GBM Cells

Cell migration and invasion were analyzed using wound-healing and transwell assays, respectively. Wound healing was conducted to determine whether MICAL2 promotes the migration of tumor cells. The average scratch distance of the NC group in U87 cells was (785.75 ± 16.51) µm, and the average distance of the MICAL2 knockdown group was (834.42 ± 17.02) µm (*p* > 0.05). Twenty-four hours after scraping of a confluent monolayer, MICAL2-knockdown cells significantly decelerated the rate of cell migration in the wound-healing assay (*p* < 0.001) (**Figures 4A, C**). We then obtained similar results in U251 cell lines (*p* < 0.05) (**Figures 4B, D**), indicating that downregulation of MICAL2 suppressed the migration of GBM cells. Transwell migration assay was used to assess the invasive capacity of GBM cells, in which we count the cells that pass through the filter. The results demonstrated increased migration capability as compared with the scrambled control cells (*p* < 0.05) in U87 cell lines, and we observed the same result (*p* < 0.05) in U251 cell lines, suggesting that downregulation of MICAL2 can promote the invasive ability of glioma cells (**Figures 4E, F**).

**Figure 3:**
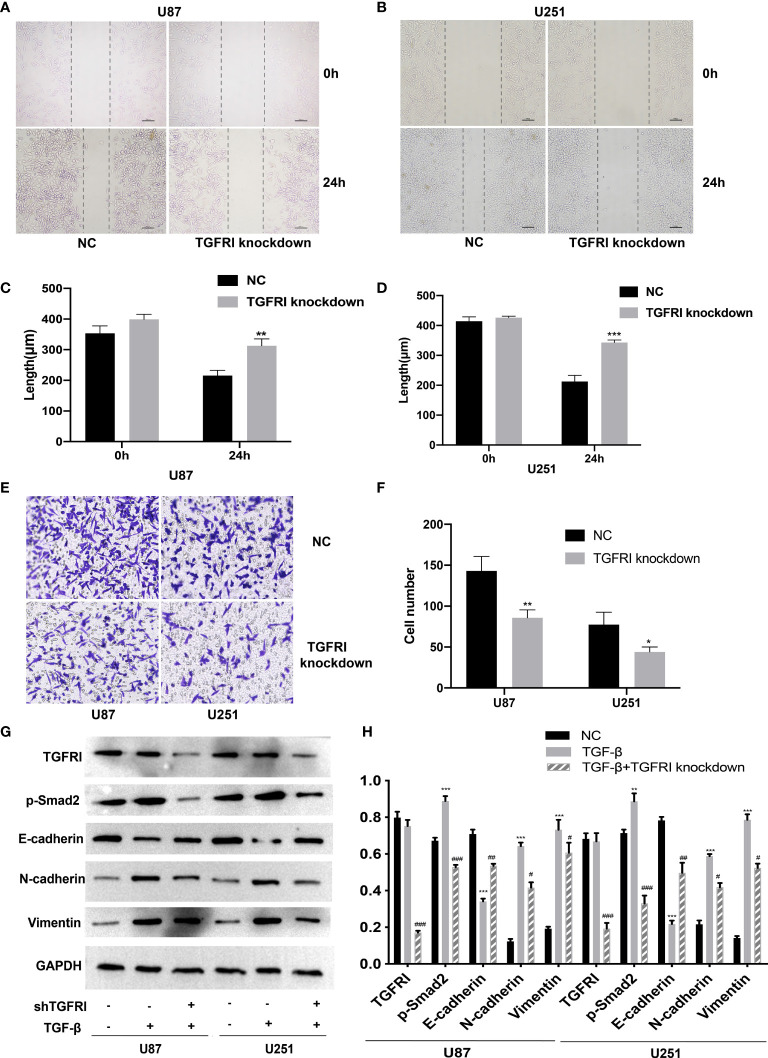
MICAL2 promotes migration and invasion of glioma cells. **(A–D)** The effect of MICAL2 on cell migration was determined *via* wound-healing assay. Comparing with the NC group, the length of wound in MICAL2 knockdown group in U87 (*p* < 0.001) and U251 (*p* < 0.05) were significantly higher after 24 h of healing. **(E, F)** Transwell migration assay was used to detected cell invasion ability of GBM cells. Cell number of MICAL2 knockdown group were decreased in U87 (*p* < 0.05) and U251 (*p* < 0.05) cell lines compared with the NC group. **(G, H)** MICAL2, p-Smad2, and EMT relative protein (E-cadherin, N-cadherin, and vimentin) expression were detected *via* Western blotting. Data are shown as the means ± SEM of three experiments. Asterisk represents TGF-β-induced group comparing with the NC group; number sign represents TGF-β-induced and MICAL2-knockdown group comparing with TGF-β-induced group; *,^#^p < 0.05, **,^##^ *p* < 0.01, ***,^###^ *p* < 0.001.

We also constructed a shRNA to knockdown TGFRI based on previous studies (36). Transwell migration and wound healing were performed in the shTGFRI group and also in the NC group. The result of transwell and wound healing revealed that the migration and invasion of U87 and U251 cells in the shTGFRI group were decreased (*p* < 0.05) (**Figures 3A–F**). It suggested that the knockdown of TGFRI could also inhibit the migration and invasion of glioma cells.

### MICAL2 Promotes EMT-Like Process of Glioma Cells Through TGFRI/p-Smad2 Signaling Pathway

**Figure 4:**
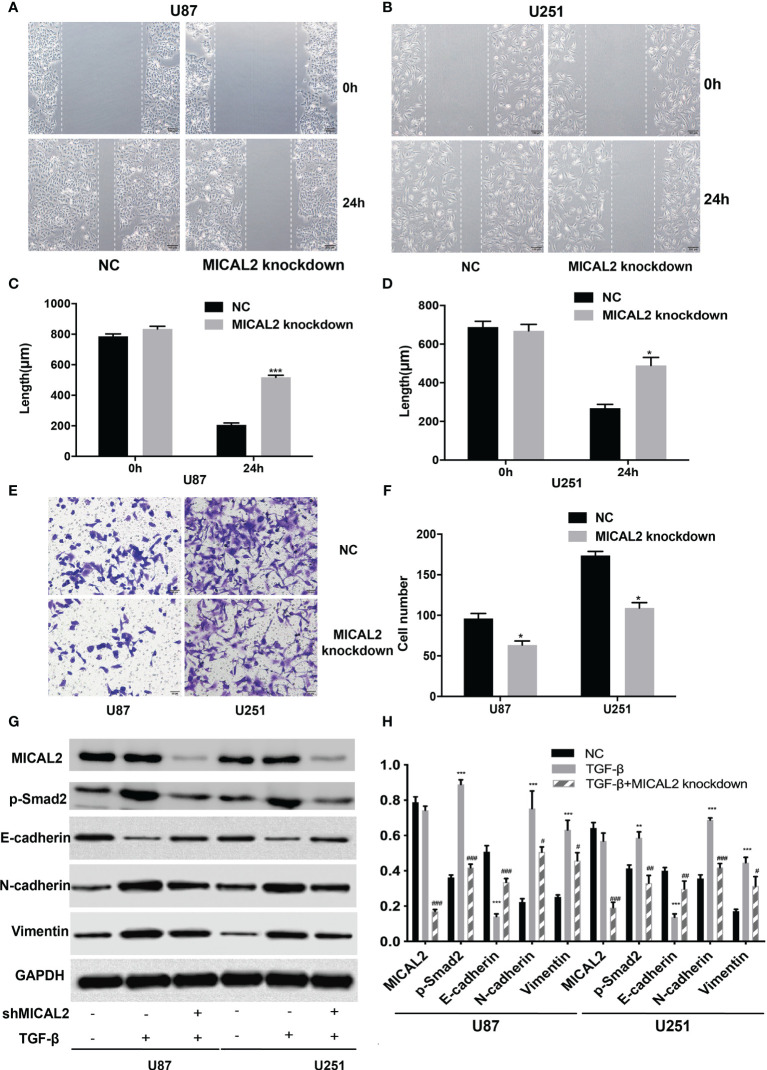
Downregulation of TGFRI supressess migration and invasion of glioma cells. **(A–D)** The effect of TGFRI on cell migration was determined *via* wound-healing assay. Comparing with the NC group, the length of wound in TGFRI knockdown group in U87 (*p* < 0.001) and U251 (*p* < 0.05) were significantly higher after 24 h of healing. **(E**, **F)** Transwell migration assay was used to detect cell invasion ability of GBM cells. Cell number of TGFRI knockdown group were decreased in U87 (*p* < 0.05) and U251 (*p* < 0.05) cell lines compared with the NC group. **(G**, **H)** TGFRI, p-Smad2, and EMT relative protein (E-cadherin, N-cadherin, and vimentin) expression were detected *via* Western blotting. Data are shown as the means ± SEM of three experiments.Asterisk represents TGF-β-induced group comparing with the NC group; number sign represents TGF-β-induced and TGFRI-knockdown group comparing with the TGF-β-induced group; *,^#^ *p* < 0.05, **,^##^ *p* < 0.01, ***,^###^ *p* < 0.001.

Recent studies showed that TGF-β can induce tumor progression and metastasis through EMT process in breast cancer (37), pancreatic ductal adenocarcinoma (38), and gastric cancer (39). Due to the different embryonic origin of the GBM cells, what occurs during GBM progression is not a complete EMT, but rather a transformation process similar to EMT (8). The EMT-like process of glioma tumor cells included the reduction of E-cadherin in addition to the rise of N-cadherin and vimentin (9). We used the TGF-β-induced glioma to simulate the EMT-like model and MICAL2 were knocked down using shMICAL2. The expression level of MICAL2 was significantly lower in the TGF-β and MICAL2 knockdown groups (*p* < 0.001), while there was no statistical difference between NC and TGF-β. The expression level of p-Smad2, the downstream molecular of TGF-β, was decreased after MICAL2 were knockdown in U87 comparing with the TGF-β group (*p* < 0.001). After the stimulation with TGF-β, a reduction in expression levels of E-cadherin and an elevation of vimentin and N-cadherin were observed both in the TGF-β-induced group. E-cadherin has increased slightly in TGF-β-induced and MICAL2-knockdown GBM cells compared with the TGF-β-induced group. Likewise, both cell lines upregulated the expression of regulators related to EMT-like process such as N-cadherin, vimentin in the MICAL2-knockdown group (**Figures 4G, H**). EMT-like process was also evaluated in the shTGFRI group (**Figures 3G, H**).

To further analyze the relationship between TGF-β and E-cadherin, N-cadherin, and vimentin, the marker proteins of EMT, we selected 16 cases of glioblastoma with the highest and lowest expression levels of MICAL2 in the TCGA database, respectively. The analysis showed that the mRNA expression level of TGF-β in the high MICAL2 expression group was strongly linearly correlated with vimentin (Pearson’s *r* * *= 0.82) and correlated with E-cadherin negatively (Pearson’s *r* = −0.42) (**Figure 6A**). In the low MICAL2 expression group, the linear correlations were poor (**Figure 6B**). As p-smad2 is a phosphorylation-modified protein, its expression level was not directly related to gene expression. Therefore, analysis was not performed on p-smad2. We also analyzed the relationship between TGFRI and E-cadherin, N-cadherin, and vimentin. The results showed that the mRNA expression of TGFRI in low MICAL2 correlated with N-cadherin (Pearson’s *r* = −0.42) and vimentin (Pearson’s *r* =  −0.48) negatively (**Figure 6C**). In high MICAL2 expression group, E-cadherin was strongly linearly correlated with vimentin (Pearson’s *r* = 0.59) and correlated with E-cadherin negatively (Pearson’s *r* = −0.39) (**Figure 6D**). The above results suggest that there may be transcriptional regulation of vimentin, N-cadherin, and E-cadherin by TGF-β in the MICAL2 high expression group.

### MICAL2 Binds With TGFRI to Interact With TGF-β

**Figure 5:**
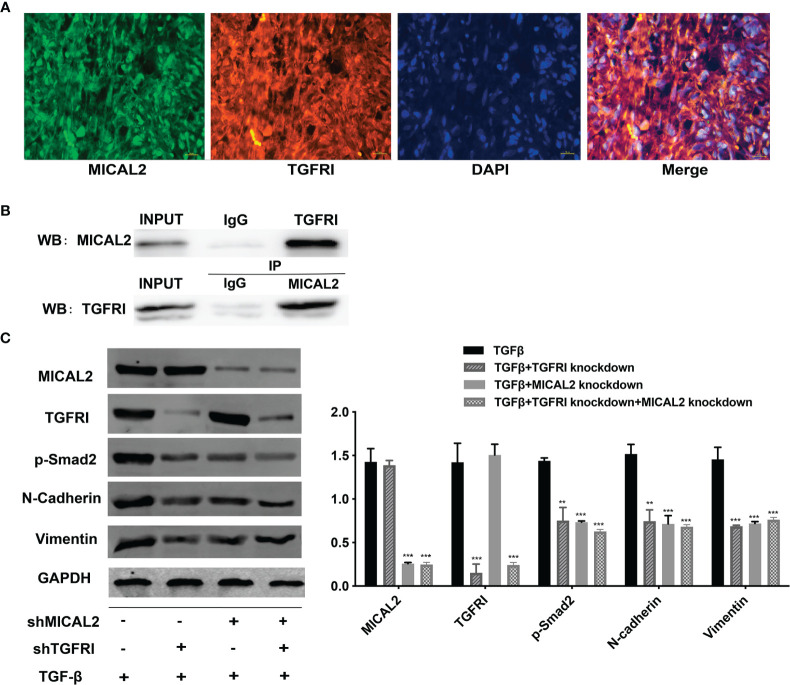
MICAL2 interacts with TGFRI. **(A)** Immunofluorescence was used to detect MICAL2 and TGFRI position in GBM cells. **(B)** Lysates of GBM cells were immunoprecipitated with anti-IgG, anti-MICAL2, or anti-TGFRI antibodies, respectively, and the products were analyzed by Western blotting with the indicated antibodies. **(C)** MICAL2, TGFRI, p-Smad2, and EMT relative protein (N-cadherin and vimentin) expression were detected *via* Western blotting. Data are shown as the means ± SEM of three experiments.Asterisk represents comparing with the TGF-β-induced group; ^**^ *p* < 0.01, ^***^ *p* < 0.001.

The above results showed that MICAL2 and TGF-β were widely expressed in GBM and high MICAL2 expression could impact the progression of patients with GBM. The classic TGF-β/Smad signaling pathways are initiated by binding the type I (TGFRI) and type II (TGFRII) receptors that are located on the membrane. To find out the role MICAL2 played in the TGF-β/Smad signaling pathways, immunofluorescences for DAPI, MICAL2, and TGFRI were performed. The immunofluorescence localization study showed that the protein MICAL2 colocalized with TGFRI, indicating that MICAL2 interacts with TGF-β through binding TGFRI (**Figure 5A**). Additionaly, co-IP also showed that MICAL2 interacted directly with TGFRI (**Figure 5B**). We detected the expression of various proteins such as MICAL2, TGFRI, p-Smad2, and EMT markers (N-cadherin and vimentin) after simultaneous transfection of shMICAL2 and shTGFRI during TGF-β induction in U87 cells to find out the interactions between MICAL2 and TGFRI (**Figures 5C, D**). The results also indicated that MICAL2 binded with TGFRI and promoted EMT-like process of glioma cells through TGFRI/p-Smad2 signaling pathway.

**Figure 6:**
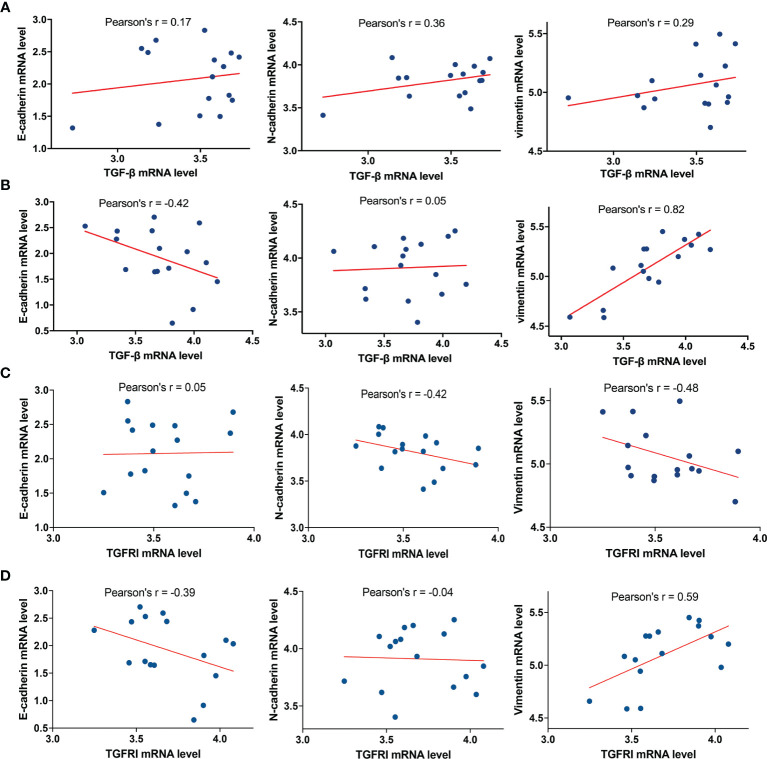
MICAL2 promotes EMT-like of glioma cells through TGFRI/p-Smad2 signaling pathway. **(A, B)** Correlation of TGF-β expression with E-cadherin, N-cadherin, and vimentin in the low MICAL2 expression group and high MICAL2 expression group. **(C, D)** Correlation of TGFRI expression with E-cadherin, N-cadherin, and vimentin in the low MICAL2 expression group and high MICAL2 expression group.

### MICAL2 Promotes the Proliferation and EMT-Like Process of GBM Cells *In Vivo*

**Figure 7:**
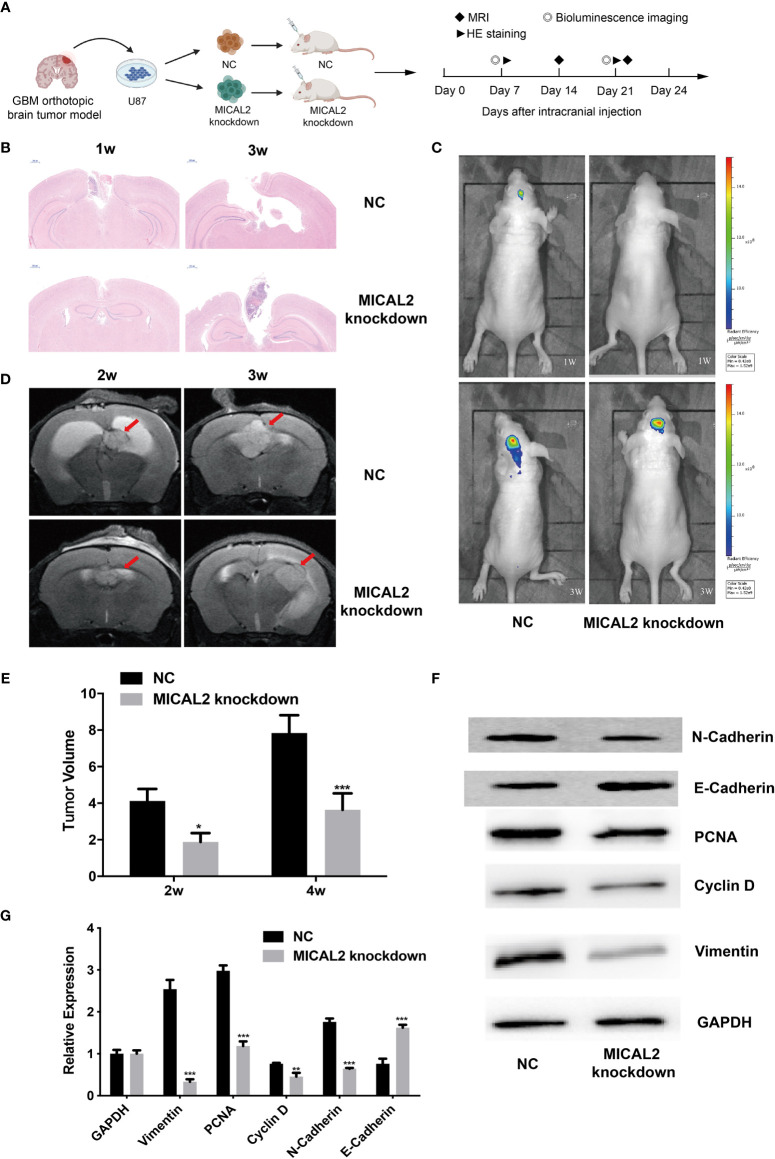
MICAL2 promotes the proliferation of glioma cells *in vivo*. **(A)** Schematic of the MICAL2 knockdown and NC in patient-derived GBM orthotopic xenograft model (created with biorender.com) (*n* = 15 for each group). **(B)** HE staining of orthotopic brain tumor. **(C)** Bioluminescence image of orthotopic brain tumor. **(D, E)** MRI image of orthotopic brain tumor, and the tumor size were analyzed. **(F, G)** PCNA, cyclin D, vimentin, N-cadherin, E-cadherin, and GAPDH expression detected by Western blotting. Data are shown as the means ± SEM of three experiments. ^*^p < 0.05, ^**^p < 0.01, ^***^p < 0.001.

To confirm whether MICLA2 promotes the proliferation of glioma in nude mice, we injected MICAL2 knockdown cells subcutaneously into the dorsal side of the mice. The tumors in the MICAL2 knockdown group grew slower compared with that of the NC group in weeks 1 and 3 (**Figure 7A**). The impacts of MICAL2 on tumor growth in an orthotopic brain tumor model were observed by MRI, bioluminescence, HE staining, and Western blotting. There is a significant difference in the dimensions of the tumor observed by MRI at the end of the study. Two weeks after injection, the tumor volume in the scramble control group was (4.12 ± 0.66) mm^3^, which was larger than the MICAL2-knockdown group (4.12 ± 0.66 mm^3^ *vs*. 1.88 ± 0.48 mm^3^, *p* < 0.05) (**Figures 7D, E**). In week 4, the average tumor volume in the scramble control group was significantly larger than that in the MICAL2 knockdown group (7.84 ± 0.97 mm^3^ *vs*. 3.64 ± 0.90 mm^3^, *p* < 0.001), which indicated that the differences between the tumor volume of the MICAL2-knockdown group and the scramble control group turned out to be bigger (**Figures 7D, E**). HE staining of the glioma tissue showed that the tumor boundary was unclear in the scramble control group and that tumor cells were invading the adjacent normal tissues both in week one and week three. On the contrary, the border of the tumor with surrounding tissues was distinct in the MICAL2-knockdown group (**Figure 7B**). Bioluminescence also showed that the tumor size of the NC group was larger than the MICAL2-knockdown group (**Figure 7C**). Furthermore, the results of Western blotting of the tumor showed that PCNA, cyclin D, and vimentin protein were substantially overexpressed in the scramble control group in comparison with the MICAL2-knockdown group (**Figures 7F, G**). PCNA was served as a DNA clamp for DNA polymerase to participating in DNA replication (40); cyclin D primarily controls the transition in mammalian cells from phase G1 to phase S, indicating that the knockdown of MICAL2 inhibited the proliferation of glioma cells (41). The expression level of vimentin and N-cadherin were decreased and E-cadherin was increased after MICAL2 was being knocked down, suggesting that the EMT-like process was inhibited. In a word, the experimental results implied that MICAL2 promotes the proliferation of glioma cells *in vivo*.

## Discussion

GBM is a common threat to human health that remains a significant problem due to the postoperative recurrence after resection, leading to poor outcomes. It is hypothesized that proliferation and metastasis are essential factors of tumor development and progression. Several signal pathways in epithelial tumors accelerating cancer metastasis, including EMT, have been widely studied. There have been disputes on whether GBM cells undergo the EMT process or not, as most GBM cells are of neuroepithelial origin. However, there is a transformation process similar to EMT in glioblastoma, expressing some characteristics of mesenchymal cells, which is called EMT-like process (42). The EMT-like process of tumor cells includes the reduction of cell adhesion proteins such as E-cadherin, and the rise in mesenchymal markers such as N-cadherin and vimentin (9), leading to reduced intercellular adhesion and loss of cell polarity and ultimately enhancing tumor invasion and migration (43). Evidence suggests that an increased expression level of TGF-β is a key factor in EMT, as cancer cells increase TGF-β signaling and become more invasive (44). Moreover, TGF-β activates Smad2 and Smad3 by binding to type I (TGFRI) and type II (TGFRII) receptors. The phosphorylated Smad2 and Smad3 form trimers with Smad4, which subsequently enter the nucleus and activate or repress transcription of several target genes, including E-cadherin, Snail, and vimentin (45).

MICAL2 is a member of molecules that interact with CasL (MICALs), a conserved family of multidomain signaling proteins. Recent studies have highlighted the relevance of MICAL in tumors. MICAL2 prostate cancer variants (MICAL2-PV) are found to be overexpressed in prostate cancer, and the protein expression of MICAL2-PV is associated with prognosis in patients with colorectal cancer (46). MICAL2 is overexpressed in bladder tumors and participates in its pathogenesis (25). MICAL2 expression is at a high level in the primary and metastatic tumor, as indicated in the EMT state (47). It has been reported that MICAL2 is a vital gene signature in the regulation of EMT, for being upregulated in mesenchymal cells (48). MICAL2 is involved in EMT, resulting in F-actin disassembly and cytoskeletal dynamics (49). Possible mechanisms are the SRF/MRTF-A signaling pathway, Sema/Plexin pathway, and through inducing ROS production (26).

At first, we found that the expression levels of TGF-β and MICAL2 were higher in high-grade glioma cells and that MICAL2 was associated with higher p-Smad2 upon evaluation, using immunohistochemistry. The high MICAL2 expression level was identified as a risk factor for overall survival in patients with primary glioblastoma. We found that MICAL2-knockdown suppressed the G1/S transition in both U87 and U251 cell lines. Then, we constructed MICAL2 knockdown shRNA and TGFRI knockdown shRNA and evaluated their effects on the ability of glioma cells to invade and migrate in U87 and U251 cell lines. We also evaluated their effects on the EMT-like process of glioma cells and found downregulation of MICAL2 and TGFRI could inhibit the EMT-like process of glioma cells. The results of the immunofluorescence localization study showed that the protein MICAL2 colocalized with TGFRI. In addition, the Co-IP results confirmed that MICAL2 interacted with TGFRI. Therefore, we infer that MICAL2 promotes the production of TGF-β downstream molecular p-Smad2 by binding with TGFRI, indicating that MICAL2 plays its role through the TGFRI signaling pathway.

Moreover, TCGA analysis revealed that there may be transcriptional regulation of vimentin and E-cadherin by TGF-β in the MICAL2 high expression group. Besides, the tumor volume in the scramble control group was larger compared with that of the MICAL2 knockdown group in both the orthotopic brain tumor model and the subcutaneous tumor model, suggesting that MICAL2 promotes the proliferation of glioma cells *in vivo*.

MICAL2 can induce F-actin depolymerization through redox modifications, decrease G-actin, and ultimately cause MRTF-A to accumulate in the nucleus, which is the downstream factor of TGF-β in the TGF-β-induced EMT (26). Moreover, MRTF-A accumulation in the nucleus activates SRF/MRTF-A-dependent gene transcription (29), upregulating cytoskeleton-associated proteins (50). There is evidence that MRTF promotes EMT in pancreatic cancer and promotes the formation of pancreatic cancer-like stem cells (51). Besides, TGF-β signaling could also observe the activation of the SRF/MRTF-A module (52). In this present study, MICAL2 promotes proliferation and migration of glioma cells through the TGF-β/EMT signaling pathway. The concrete mechanism is still unknown, but the SRF/MRTF-A may have a close connection with the formation of MICAL2-related EMT. Therefore, the MICAL2 downstream gene and the detailed mechanism of EMT should be further investigated.

## Conclusion

In conclusion, our study clarified that MICAL2 interacts with TGFRI, and MICAL2 is associated with higher p-Smad2. High MICAL2 expression could be identified as a risk factor for overall survival in patients with primary glioblastoma. The results confirmed that the downregulation of MICAL2 in GBM would inhibit proliferation and migration of glioma cells and have explained the signaling pathway behind it. In addition, our experiment indicates that the detailed mechanism working in MICAL2 is not clear and should be further investigated. In the future, understanding the molecular pathways involved in MICAL2 will help develop new targeted therapies for glioblastoma and improve currently available therapies.

## Data Availability Statement

The raw data supporting the conclusions of this article will be made available by the authors, without undue reservation.

## Ethics Statement

The animal study was reviewed and approved by the Animal Experimentation Ethics Committee of Wuhan University.

## Author Contributions

BP, XX, and YL contributed to the conception of the study and wrote the manuscript. XZ and OM performed the experiment. XZ contributed significantly to analysis and manuscript preparation. GL and ZJ performed the data analyses. LG helped perform the analysis with constructive discussions. XX and NZ supervised the project. All authors contributed to the article and approved the submitted version.

## Funding

This study was supported by Wuhan Young and Middle-aged Medical Backbone Talents Training Project in 2017 to Zou Ning.

## Conflict of Interest

The authors declare that the research was conducted in the absence of any commercial or financial relationships that could be construed as a potential conflict of interest.

## Publisher’s Note

All claims expressed in this article are solely those of the authors and do not necessarily represent those of their affiliated organizations, or those of the publisher, the editors and the reviewers. Any product that may be evaluated in this article, or claim that may be made by its manufacturer, is not guaranteed or endorsed by the publisher.

## Abbreviations

MICAL2, molecule interacting with CasL2; TGF-β, transforming growth factor beta; TGFRI, TGF receptor-type I; EMT, epithelial-to-mesenchymal transition; GEPIA, gene expression profiling interactive analysis; TCGA, The Cancer Genome Atlas; GBM, glioblastoma multiforme; WHO, World Health Organization; IDH1, isocitrate dehydrogenase 1; CSCs, cancer stem cells; SRFs, serum response factors; MRTFs, myocardin-related transcription factors; DMEM, Dulbecco’s modified Eagle’s medium; MICAL2-PV, MICAL2 prostate cancer variants.
