# Supplementary material for: MICAL2 Promotes Proliferation and Migration of Glioblastoma Cells Through TGF-β/p-Smad2/EMT-Like Signaling Pathway
Source: Front Oncol. 2021 Nov 12;11:735180. doi: 10.3389/fonc.2021.735180 (PMC8632809; doi:10.3389/fonc.2021.735180)
Supplement: Supplementary file 1 [file DataSheet_1.docx]

**Supplementary materials**

Figure S1: The level of the indicated proteins in GBM cells with MICAL2 knockdown for 0h, 24h and 48h.


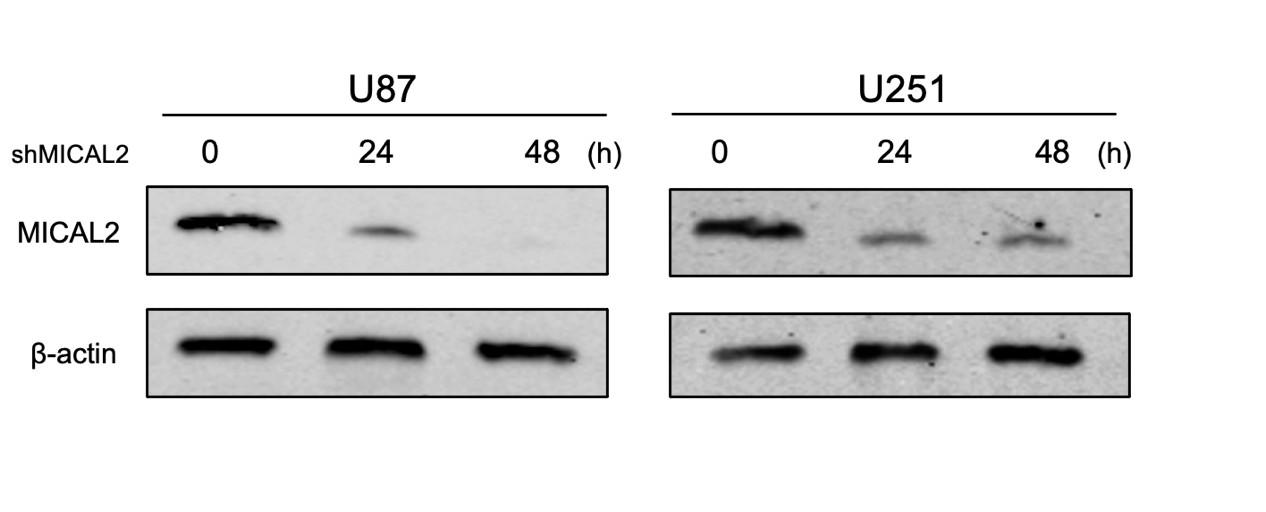


Figure S2: The expression level of TGFRI after transfection with shTGFRI was detected using RT-PCR.


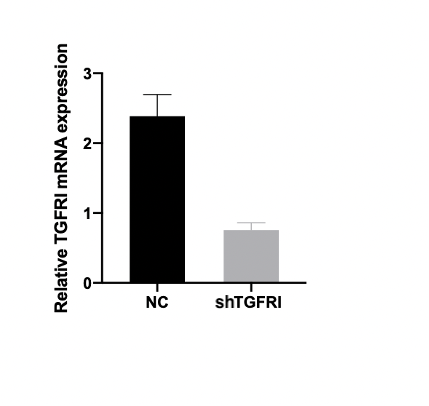


Figure S3: The expression level of MICAL2 and p-Smad2 after transfection with shMICAL2#2 was detected using Western blot.


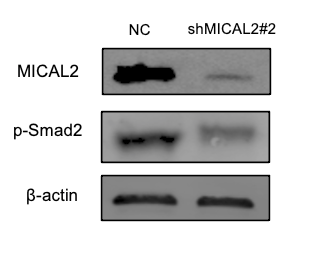


**Table S1**

| shMICAL2#1 | 5’-GCUGGGAGUUGAAAUCCAUTT-3’ |
| --- | --- |
| shMICAL2#2 | 5’-TCACTTCATTCACTGTAAA-3’ |
| shTGFRI | 5′-GACAUCUAUGCAAUGGGCUUAGUAU-3′ |

**Table S2**

Primer sequences for RT-qPCR

|  | Forward (5’-3’) | Reverse (5’-3’) |
| --- | --- | --- |
| TGFRI | CATCGCCTCAGACATGACCTC | GTTTGCCCTGTGTACCGAAGA |
| GAPDH | CTGGGCTACACTGAGCACC | AAGTGGTCGTTGAGGGCAATG |
