# Supplementary figures and images for: MICAL2 Promotes Proliferation and Migration of Glioblastoma Cells Through TGF-β/p-Smad2/EMT-Like Signaling Pathway
Source: Front Oncol. 2021 Nov 12;11:735180. doi: 10.3389/fonc.2021.735180 (PMC8632809; doi:10.3389/fonc.2021.735180)

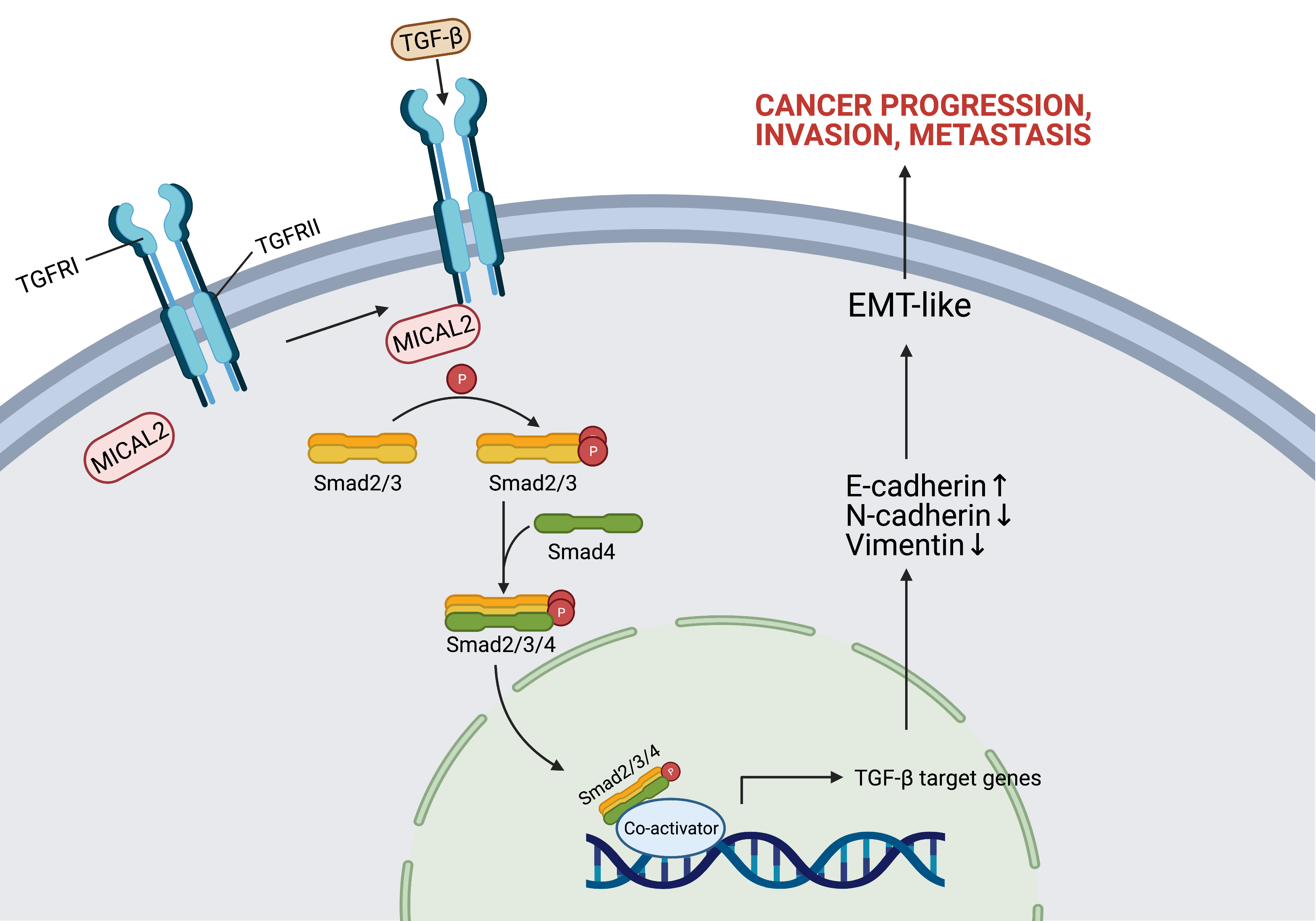

Supplement: Supplementary file 2 [file Image_1.png]
